# Facilitators and Barriers to Access to Midwife‐Led Birth Settings for Racialized Women in the UK: A Scoping Review

**DOI:** 10.1111/birt.12897

**Published:** 2024-12-09

**Authors:** Anna Melamed, Lucia Rocca‐Ihenacho, Anna Horn, Christine McCourt, Frances Rivers, Marina Alice Sylvia Daniele

**Affiliations:** ^1^ City University of London London UK; ^2^ University of the West of England Bristol UK; ^3^ Kingston Hospital NHS Foundation Trust Kingston UK

**Keywords:** BAME, birth centre, black women, ethnic minority, home birth, midwifery unit, racism

## Abstract

**Background:**

In UK maternity care, racialized women have worse experiences and clinical outcomes than White women. Midwife‐led birth settings (MLBS), including home births and midwife‐led units, both freestanding and alongside hospitals, are all available as choices for low‐risk women in the UK. MLBS deliver optimal outcomes for low‐risk women with uncomplicated pregnancies, including for racialized women, and can offer culturally specific care, possibly mitigating existing social inequalities. Evidence suggests that racialized women access MLBS less than White women.

**Aim:**

To map existing literature on facilitators and barriers to accessing MLBS for racialized women and to identify emerging themes.

**Method:**

A scoping review of UK literature over the last 10 years using OVID, Ebsco Host, and gray literature. Search, selection, and data extraction were performed using PRISMA and JBI guidelines. Data were analyzed using inductive thematic analysis.

**Results:**

Fourteen articles met the inclusion criteria, only one addressing the research question directly and others containing some relevant material. Six themes were identified: admission criteria, information giving, the role of antenatal groups, bias and assumptions, beliefs about birth, and MLBS as empowering.

**Conclusions:**

There is a lack of research on racialized women's access to MLBS. Community outreach, having midwifery services embedded in the community, defaulting to MLBS for women categorized as low risk, continuity of carer, and interventions achieving a reduction in care‐giver bias may improve access and outcomes.

## Graphical abstract

A scoping review examining racialised women's access to midwife led birth settings found assumptions about preferred birth place being made by midwives, women and their families and differences in information given. Group antenatal care, inclusive representation, and opt‐out, flexible admission criteria may improve access and ultimately help mitigate inequalities.

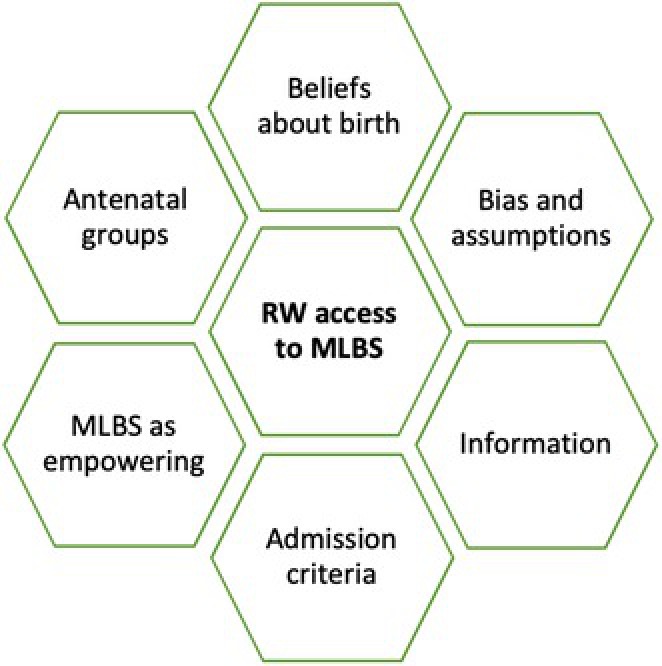

## 1 Definitions

We use the term ‘racialized women’ to encompass maternity service users who are not White and who are racialized by UK society. Where relevant or for the veracity of reporting, we use the study authors' terms such as Black, Asian, and minority ethnic (BAME). We acknowledge that not all those who get pregnant identify as women. In our review, we use the word woman throughout, as this is the term used in all the studies. In the discussion, this should be taken to include people who do not identify as women but who are pregnant or giving birth.

Midwife‐led birth settings (MLBS) refer to the home and midwifery units or birth centers, both alongside hospitals' obstetric units and freestanding. In these settings, midwives take primary professional responsibility and practice a midwifery model of care [1, 2]. Access means not just the supply of services but the extent to which women can utilize them and how acceptable they are, and may depend on organizational, social, or cultural factors [3].

As authors, we identify as two White British, one White Irish, one Black American, and two White Italian. Four of us are midwives, all of whom have all worked with racialized women accessing MLBS. All of us currently live in the UK and variously have Jewish and Irish heritage, are migrants, or live in mixed‐race families. We have all brought our own perspectives, both insider and outsider, of different facets of this issue.

## 2 Background

### 2.1 Maternity Outcomes and Ethnicity

Racialized women in the UK have a higher likelihood of suffering inequality, including lower economic status [4], practical and psychological stress due to racist migration laws [5], social and cultural inequalities [6], including health inequality and institutional racism [7, 8, 9, 10]. Over time, the persistent, repeated, unceasing nature of these onslaughts can accumulate and become a cause of poor health in a process described as ‘weathering’ [11]. The UK has a well‐established midwifery service and access to obstetric care, free at the point of use. Despite this, Black women in the UK are still four times more likely to die in the perinatal period [12], and babies born to Black women are up to twice as likely to die. The 2021 UK Maternity Audit reported an overall caesarean rate of 33% for Black women and 25% for White women [13]. However, the data does not show us if this is due to a difference in morbidities or a difference in care. Research on racialized women using UK maternity services consistently cites direct and indirect racism, such as not being listened to or respected, hearing racially discriminatory language, and assumptions being made about education level or background, pain tolerance, and behavior in labor [14, 15, 16]. There is a reported lack of knowledge among midwives about culture and about physiology (such as presentation of clinical conditions on darker skin) [16, 17]. This can have an impact on access, as a mistrust of services can lead to some women withdrawing from care [18].

Research into migrant women in the UK and pregnant women seeking asylum in comparable high‐income countries has an overlap with our population of interest as a significant proportion of migrant women are racialized [17, 19]. Research revealed that they felt isolated, ignored, and alone. Other reported barriers to access for migrant women include not being aware of the specificities of the NHS maternity system, insufficient translation or interpreting services for those with limited English, and a lack of money for travel to appointments [17, 20].

### 2.2 Benefits of Midwife‐Led Birth Settings for Racialized Women

For healthy women with uncomplicated pregnancies, MLBS compared to obstetric units have lower rates of caesarean or instrumental birth and postpartum hemorrhage, better breastfeeding rates, reduced medium‐ and long‐term maternal morbidities, no difference in neonatal outcomes [21, 22, 23, 24, 25], and higher levels of maternal satisfaction [26, 27, 28]. Secondary data from the Birthplace Study showed both racialized women and White women had an equally reduced chance of intervention such as instrumental deliveries in MLBS compared to obstetric units [29]. The community‐based Albany Midwifery Practice had high rates of MLBS (34% home birth rate) for racialized women and notably better maternal and neonatal outcomes for racialized women and their babies than contemporary national averages [30].

The midwifery model of care can offer highly personalized, woman‐centered relational care and the possibility of continuity of carer [6, 30, 31, 32]. MLBS are better placed than obstetric‐led settings to offer culturally safe care embedded in the communities of women they serve. There are reports of the beneficial effect of midwife care for racialized women specifically, such as *‘knowing there is someone who cares for you*’, [19] (p531) and woman‐centered continuity of care models resulting in positive experiences [33, 34, 35, 36].

UK research into midwives' views showed a will to mitigate systemic inequality and gain cultural competencies needed to care adequately for a diverse population [20]. Midwives' autonomy and the centrality of the midwife‐mother relationship increase the chance of women being listened to and respected, at best acting as a restorative force against the backdrop of racism and weathering [37, 38].

### 2.3 Midwife‐Led Birth Settings and Access

Only 15% of women in the general population in England access MLBS [39, 40] despite an estimated 45% being eligible for MLBS at the start of labour [41, 42]. Research into access and utilization of MLBS falls into themes of organizational factors, midwives' influence, and women's culture and beliefs. Organizational barriers include a lack of service provision [43, 44], inconsistent service provision caused by short staffing [45], lack of commitment by providers to regard MLBS as a core part of the service, perceiving it instead as an optional add‐on [42], the depth of the culture of medicalization, the construction of birth as inherently risky [46, 47], fears of litigation (realistic or otherwise) [42], and an us and them attitude between obstetric unit staff and MLBS midwives [42]. Women may face challenges with admission in early labour [45] and find it logistically easier to opt for birth in an obstetric unit rather than MLBS [46]. Midwives' own preferences, biases, and attitudes regarding risk show some seeing freestanding midwifery‐led units as being less safe and less popular with women [42]. This affects the information they give, and thus women's decision making [42, 43, 44, 46].

### 2.4 Racialized Women's Access to Midwife‐Led Birth Settings

There is evidence that rates of MLBS use are even lower for racialized women. The Birthplace study revealed a higher proportion of affluent White women accessing freestanding midwifery units and home births and shows that of women starting labor in MLBS, 89% were White and 11% racialized women, compared to women biomedically classified as low‐risk starting labor in the obstetric units at 82% White and 18% racialized women [21]. A study on women biomedically classified as low‐risk who had waterbirths, which are vastly more common in MLBS, showed Black and Asian women were less likely to have a waterbirth at 6% and 4%, respectively, compared to 15% of White women [48]. Henderson et al. [49] analyzed data from a survey of over 24,000 women in England collected in 2010. They report that 6.7% of White women respondents accessed MLBS, but significantly fewer Pakistani (4.2%) and Black African women (2.7%) used them. Waterbirth rates for Pakistani (0.2%), Indian (1.9%), Bangladeshi (1.6%), and Black African (2.2%) women were significantly lower than for White women (5.2%). In Tower Hamlets, London, the home birth team showed 29% of its small caseload of 59 women in 2018 to be from ‘BAME’ backgrounds compared to 55% of the local population. However, a well‐established MLU in the same borough achieved a higher proportion of women from Black and South Asian backgrounds, arguably as a result of extensive community outreach and a continuity of care model [36, 50]. Research in the US showed race being the single most important factor for the rate of transfer from midwife‐led to obstetric care, even when adjusted for other variables, possibly due to provider bias (being quicker to refer) or poor provider‐patient communication [51].

## 3 Objective

This scoping review examined the literature on facilitators and barriers to access to MLBS for racialized women in the UK.

## 4 Methods

We followed JBI scoping review guidelines [52, 53] and registered a protocol developed with the team researching accessibility of MLBS in the UK to racialized people [54, 55]. A scoping review was chosen as the most appropriate method for the identification, mapping, and summary of the existing literature, allowing for inclusion of articles with other main focuses, differing methodologies, and gray literature [56].

The inclusion criteria were: UK‐only research due to the unique racial history and specific context of NHS midwife‐led services; conducted within the last 10 years to reflect the contemporary situation; and academic and gray literature to decrease any systemic (racial) bias in academic publishing and increase the possibility of including grassroots‐produced material, although in fact none were identified. Due to the paucity of data on the subject, we included texts with only a brief reference to our topic.

**FIGURE 1:**
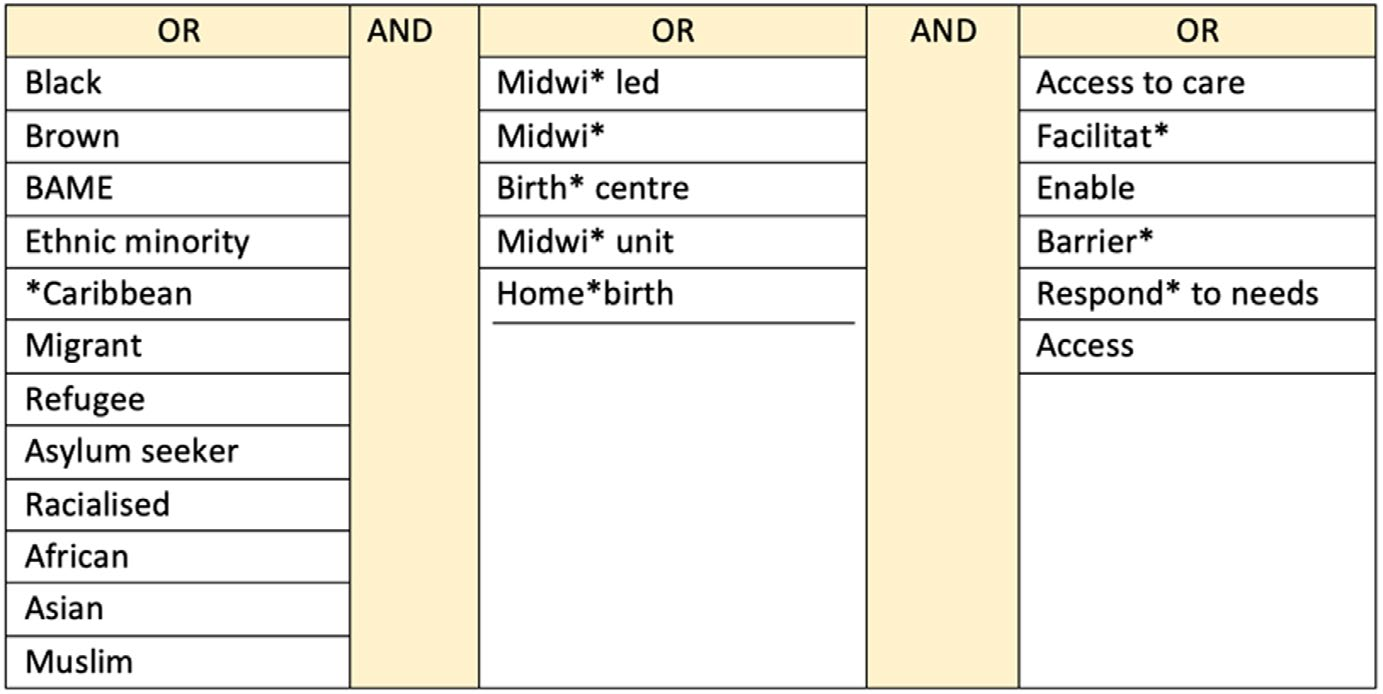
Search terms. [Colour figure can be viewed at wileyonlinelibrary.com]

Databases CINAHL and Medline Complete were searched using the EBSCO Host platform, and EMB Reviews, Embase, Global Health, MIDIRS, and Social Policy and Practice via the OVID platform. Searches were performed in January, March, and April 2023 (see Figure 1). Further literature was identified using back‐chaining, gray literature searches (City University of London Library, Gray Matters, NHS England and Gov.uk), and professional networks.

**FIGURE 2:**
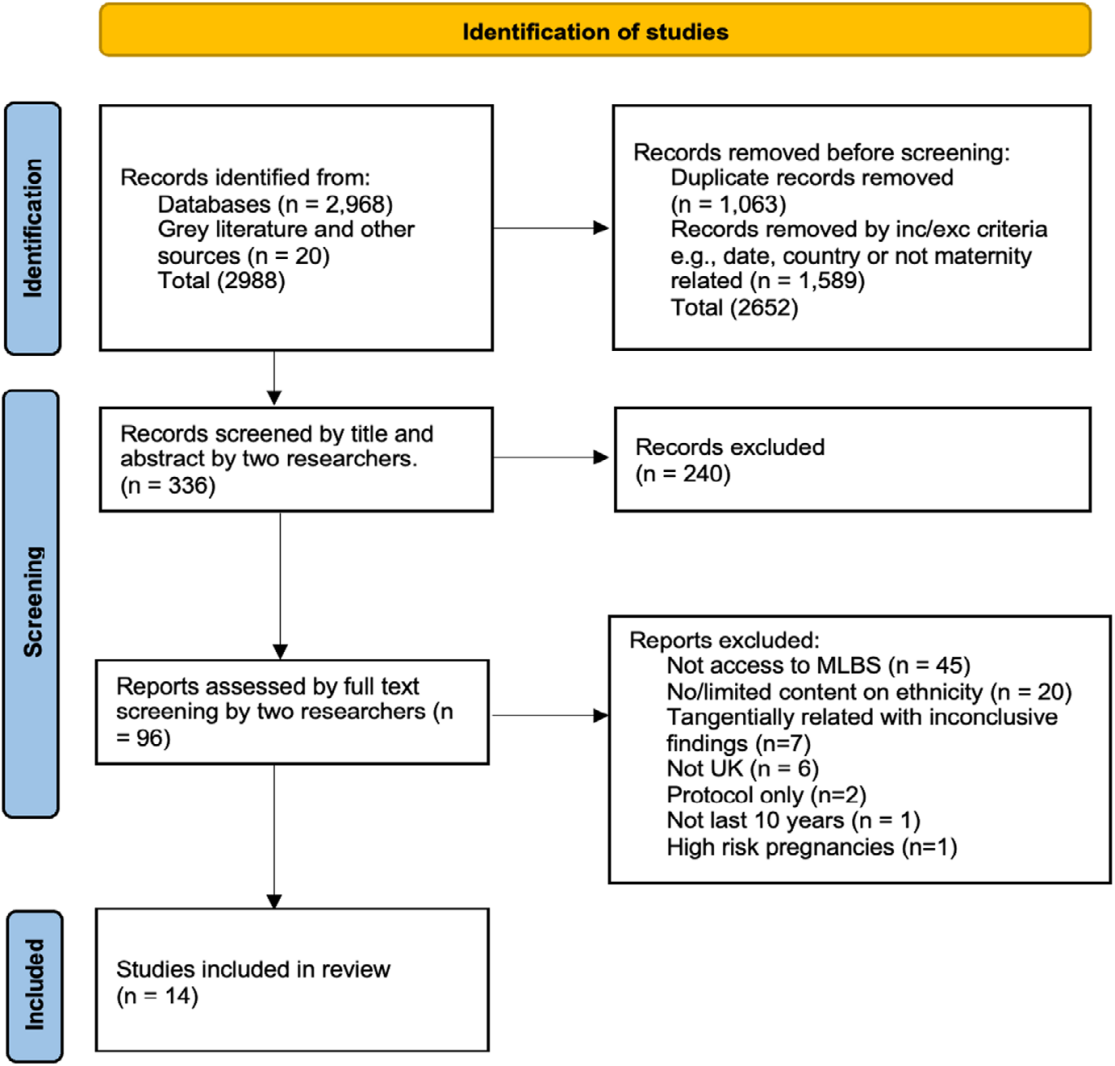
Prisma diagram. [Colour figure can be viewed at wileyonlinelibrary.com]

After duplicate removal and application of inclusion and exclusion criteria, 336 articles were selected for screening. Two researchers screened independently by title and abstract. Discrepancies were resolved through discussion, resulting in 96 articles for full‐text screening. A total of 14 articles containing relevant material were selected for inclusion in the review (see Figure 2). Data were extracted using a bespoke data extraction form primarily by one researcher, with oversight by a second. We applied the method‐appropriate CASP critical appraisal checklist. This aided rigorous analysis and ensured the methodology and quality of each study were fully considered [52, 53]. All 14 articles demonstrated sound methodological quality, lending trustworthiness to our review [57]. We performed inductive thematic analysis adapted from the method described by Thomas and Harden with the aim of thematic summary and analysis, but not thematic synthesis, as this is beyond the remit of a scoping review [52, 58].

### 4.1 Summary of Results

**FIGURE 3:**
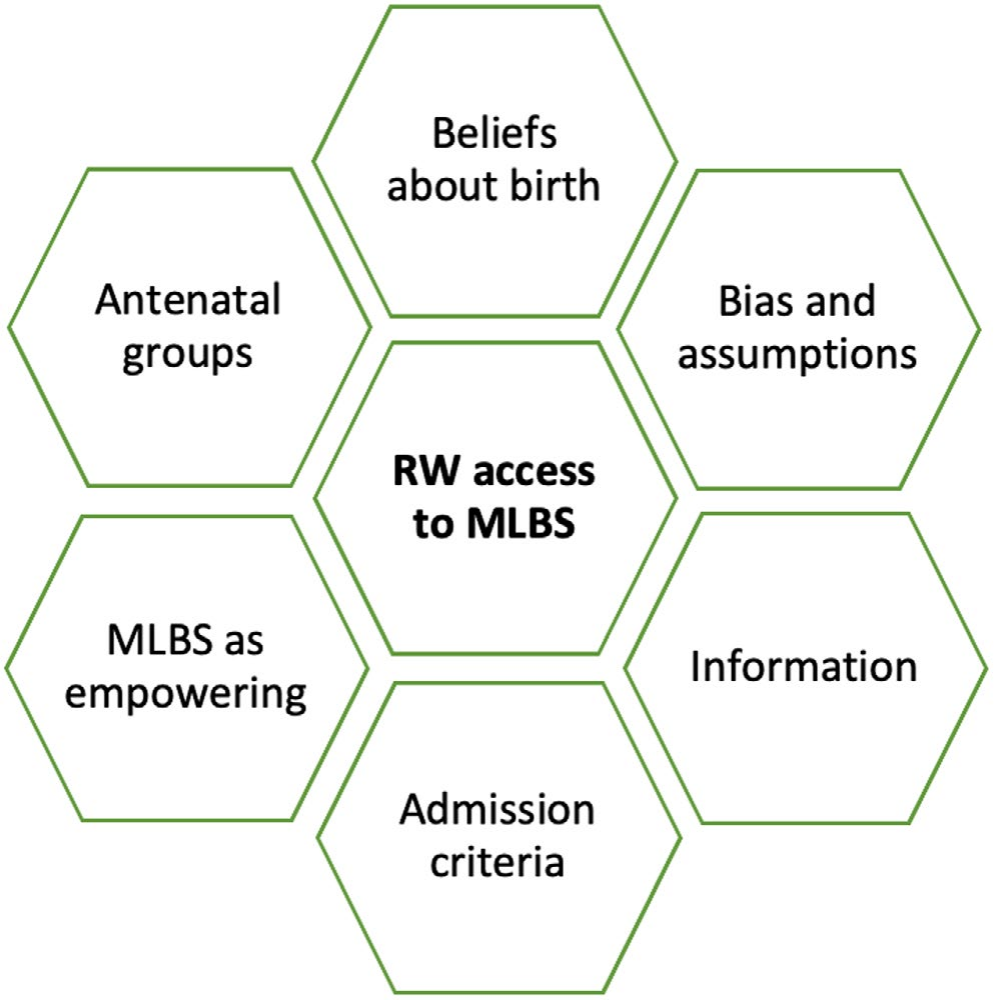
Thematic diagram. [Colour figure can be viewed at wileyonlinelibrary.com]

Fourteen texts had content addressing our question: two systematic reviews (treated as texts in their own right), eight qualitative studies, one mixed‐methods study, two audits, and one quantitative study (see Table 1). A significant finding was the lack of literature addressing the question of access and utilization of MLBS by racialized women (Figure 3). Only one article, Reeve Jones [59], addressed the research question directly. Of the other 13 studies, most addressed our question as a minor point in the context of studies on place of birth that did not focus specifically on racialized women [30, 45, 46, 50, 60, 61, 62] or studies on racialized women regarding outcomes or experience that do not focus specifically on MLBS or place of birth [33, 49, 63, 64, 65]. In the thematic summary below, only the small amount of text directly relating to the review topic is referred to.

**TABLE 1:** Selected articles summary table.

Authors, (date) [reference number]	Title	Research design	Sample and question	Data from	Key findings	CASP score/10	Proportion of text directly relevant to our question (%)
Coxon, Sandall and Fulop (2014) [67]	To what extent are women free to choose where to give birth? How discourses of risk, blame and responsibility influence birth place decisions	Qualitative, longitudinal narrative study from 3 maternity units	Women of diverse, class, race, urban/rural Views on birth place choice	2009–2010	Many African background woman see hospital as safer than home birth. Obstetric unit as default	9	4.4
Foley, Callaghan and Olusile (2019) [51]	Creating a dedicated home birth team in Tower Hamlets: a review of outcomes from the 1st year	Audit and evaluation of data from homebirth team	Data on 90 women re referral, birth place, transfer and outcomes	2018	Proportionally fewer Bengali women referred or accepted to homebirth team. Reasons: housing, midwife bias, lack of time at antenatal appointments. Need for community outreach.	9	6.9
Goodwin, Hunter and Jones (2018) [67]	The midwife‐woman relationship in a South Wales community: Experiences of midwives and migrant Pakistani women in early pregnancy	Ethnographic qualitative Semi structured interviews and observation	Nine pakistani women, 11 Midwives on relation and antenatal care	2015	Influence of Pakistani culture. Some lack of confidence in midwives. Women and midwives have different expectation of maternity care	10	17.3
Henderson, Gao and Redshaw (2013) [49]	Experiencing maternity care: the care received and perception of women from different ethnic groups	Quantitative. Statistical secondary analysis	> 24,300 women (15% not white) on experience of maternity care	2010	Women in all minority ethnic groups had a poorer experience of maternity services than White women including lack of choice	9	2.8
Henshall, Taylor, Goodwin, Farre, Jones and Kenyon (2018) [61]	Improving the quality and content of midwives’ discussions with low‐risk women about their options for place of birth: Co‐production and evaluation of an intervention package	Mixed method study on service improvement Qualitative, focus groups	10 focus groups of 38 midwives about service improvement impact	2015–2016	Midwife bias apparent in information on options and depth of conversation re MLBS according to cultural assumptions	10	5.2
Homer, Leap, Edwards and Sandall (2017) [30]	Midwifery continuity of carer in an area of high socioeconomic disadvantage in London: A retrospective analysis of Albany Midwifery Practice outcomes using routine data (1997–2009)	Retrospective analysis of existing data set. Audit of data collected by homebirth team. Service evaluation.	2568 women booked with Albany community midwifery caseload service (total cohort)	1997–2009	Importance of case‐loading community‐based midwifery. High homebrith rate. Homebirth seen as positive and normal within the community. Birth place option left open	10	2.9
Hunter, Da Motta, McCourt, Wiseman, Rayment, Haora, Wigginsa and Harden (2019) [66]	Better together: A qualitative exploration of women's perceptions and experiences of group antenatal care	Qualitative. Focus groups and semistructured interviews	26 women before and after group antenatal care	2014, 2015 and 2017	Group antenatal care as empowering. Better relations with midwives. Expanded horizons. Place of birth decision after full discussion. MLBS as option	10	11.2
Jomeen and Redshaw (2013) [63]	Ethnic minority women's experience of maternity services in England	Qualitative. Post questionnaire	219 Black and minority ethnic women	2012	Hospital perceived as safe place. Bias and racism of midwives. Lack of care overall	9	1.9
Khan (2021) [65]	Ethnic health inequalities in the UK's maternity services	Systematic review of UK studies	Eight papers (3 same as this study)	Pub: 2013–2018	Maternity services and systems. Communication and midwife‐woman relationship sometimes poor	9	5.7
MacLellan, Collins, Myatt, Pope, Knighton and Rai (2022) [33]	Black, Asian and minority ethnic women's experiences of maternity services in the UK: A qualitative evidence synthesis	Systematic review with qualitative evidence synthesis	24 papers (2 same as this study)	Pub: 2000–2021	Lack of flexibly. Rushed, one size fits all, antenatal care and place of birth discussion. Lack of continuity of carer and trust. Lack of control in decision making	9	13.4
McCourt; Rayment; Rance and Snadall (2014) [60]	An ethnographic organizational study of alongside midwifery units: a follow‐on study from the Birthplace in England programme. Chapter 5. Women and partners’ experiences and perspectives	Qualitative, observation and interviews	35 women. 12 Birth partners. (12 BAME) about access to MLU	2011–2012	Information on place of birth from friends, unit tours, antenatal classes. Etc (not midwife). Cultural assumptions by community midwife. Perceived as a luxury	10	6.9
Naylor Smith, Taylor, Shaw, Hewison and Kenyon (2018) [62]	‘I didn't think you were allowed that, they didn't mention that.’ A qualitative study exploring women's perceptions of home birth	Qualitative. Focus groups	28 women in 5 focus groups. Many ethnic minority gorups. On NHS homebirth service	2014	Assumption of obstetric unit by women. Lack of information on home birth. When option is made clear many Black women choose homebirth	9	11.2
Rayment, Rance, McCourt and Sandall (2019) [45]	Barriers to women's access to alongside midwifery units in England	Qualitative. Observation and interviews	Observations (> 100). staff interviews (*n*=89). Women and partners interviews (*n*=47)	2011–2012	Barriers: 1. when choosing MLBS. 2. Early labour. Advantage of opt‐in vs. opt‐out. Cultural assumptions by community midwife	10	9.1
Reeve Jones (2022) [59]	An ethnographic study of an urban freestanding birth centre with focus on the increase of Bengali women choosing the Birth Centre as place of birth	Ethnographic. Qualitative. Service audit and semi structured interviews	Audit: All women birthing at MLU. Interviews: Bengali women who had birthed at MLU	2021	Generational change—UK‐born women choosing the MLU. Sisterhood and kinship—the importance of knowing others who had used MLU. Reproductive agency and choice. Birth stories and representation	10	100.0

### 4.2 Thematic Summary

We identified six interrelated themes from the limited material related to racialized women's access to MLBS (Figure 3). None of the papers emphasized practical barriers such as transport or (lack of) access to free NHS care.

### 4.3 Admission Criteria and Guidelines

The initial barrier to MLBS is categorizing women as ‘high‐risk’, occurring at any time in the pregnancy, labor, and birth journey [60]. This may disproportionately disadvantage racialized women as a higher proportion of racialized women may fall outside of the biomedical low‐risk category (e.g., a higher rate of the pre‐existing comorbidities of diabetes and hypertension is found in Black and South Asian women in Great Britain) [13]. More research needs to be done on this subject to investigate the reasons for this [51]. Jomeen and Redshaw [63] interviewed a UK‐born Black Caribbean woman who was encouraged to choose the obstetric unit over home birth due to being a grand‐multiparous woman, which she felt to be discriminatory. Women interviewed by Reeve Jones [59] attempted to stay ‘low risk’ by managing their BMI or diabetes, for example. Naylor Smith et al. [62] revealed some White study participants but no racialized participants who exercised agency by changing their place of birth to access care outside their trust guidelines. However, after attending group antenatal care, some racialized women made active decisions to stay in midwife‐led care, including those with intermediate risk factors where obstetric care was offered [66].

### 4.4 Information

The assumption that women would be using the obstetric unit, an automatic referral to an obstetric unit, and lack of information about place of birth options were reported in most studies [45, 49, 60, 62, 67]. Women who sought information from informal networks, work colleagues, internet research, social media, or private antenatal classes were more likely to see MLBS as a viable option [45, 59, 60]. MacLellan [33] reported that some women were unaware of place of birth choices such as home birth, and a large 2014 survey showed a third of the women were only aware of the obstetric unit [61]. Naylor Smith et al. [62] quote: I think I was aware of home birth as an option, but certainly not from a health care professional. (p7).

Rayment et al. [45] explain that only after women had opted‐in to the MLU did they receive full antenatal information regarding the MLU. Racialized women in Naylor Smith et al.'s [62] focus groups were initially less aware of the range of choices and less likely to make active place of birth choices than White women; however, once made aware, there was an interest in MLBS. Rayment et al. [45] quote, I didn't know [AMU] was there. I just thought I would go the Labour Ward bit. But when I found out I could go to [AMU] I was like, oh great [laughter], that's much better. (p82). Homer et al. [30] and Foley et al. [50] expressed the importance of outreach and visibility of MLBS. McCourt et al. [60] concluded that an ‘opt‐out’ system for MLUs might reduce disparity of access by establishing it as the normal pathway for all ‐low risk women. Women with the Albany Midwifery Practice did not make a fixed place of birth choice in pregnancy but rather kept the final decision about place of birth an open question until labor onset [30].

### 4.5 Antenatal Classes and Groups

Reeve Jones [59] noted the importance of antenatal classes for information and confidence building: Active birth classes were fundamental to most of my respondents in terms of decision making and getting their husbands or birth companions on board. (p23). However, Henderson et al. [49] and MacLellan et al. [33] found that racialized women were significantly less likely to attend antenatal classes or be directed to them, in line with earlier studies [68].

Group antenatal care can redress imbalances by relocating knowledge of pregnancy and birth back to the women through self‐checks and discussions. Hunter et al. [69] found it shifted the dynamic away from the passive patient role that abdicates decisions to medical authority (potentially leading to obstetric unit birth) and pregnancy and birth from a medical to a social occurrence (potentially leading to MLBS as an option). It also helped those with limited English, as women helped each other express their questions or comments. The discussions helped women challenge accepted norms by talking with those outside their immediate communities (rare for some of them), normalizing the choice of MLBS [66].

### 4.6 Bias and Assumptions

Lack of control, feeling like a task to be rushed, and overly standardized care were highlighted in almost all the papers. Racialized women, particularly, are left uninformed with little time to discuss place of birth [33, 50]. Issues such as language barriers, cultural differences, or social complexities cannot be resolved in a rushed, overstretched service, leading to direct and indirect discrimination [33, 65]. Henderson et al. [49] found that racialized women were significantly less likely to report being given understandable information, involved in decision‐making, or given a choice regarding place of birth.

Both midwives and women had assumptions about ethnicity and place of birth. MLBS and water births were referred to as ‘hippy’ or for White women by those interviewed by Reeve Jones, Hunter et al. and Naylor Smith et al. [59, 62, 66] Foley et al. [50] cite the proportionally low rate of midwife referrals for homebirth for Bengali women. Many midwives shaped their discussion about place of birth based on cultural assumptions, restricting genuine choice [45, 50, 60, 61, 62]. These assumptions include that a ‘type’ of woman chooses home birth, that birth environment is only important to White middle‐class women, or that women's social relationships, home environments, and socio‐demographic variation would make them more or less likely to choose a MLBS [45].

Reeve Jones [59] and Naylor Smith et al. [62] found that discussing place of birth at each opportunity aided informed decision‐making and choice for MLBS, implying a lack of discussion may mean women are missing out. White women, however, did not shift their opinion during focus group discussions led by Naylor Smith et al. [62] indicating that more discussion might be particularly important for racialized women's access to MLBS. Racialized women accessing antenatal care later in pregnancy and engaging less may decrease the opportunities to discuss place of birth [49]. However, this pattern may result from experiencing racism in healthcare settings or a lack of understanding of the NHS maternity care system [64]. The Albany Practice normalized home birth within the community, and it became a popular option across the class and race spectrum [30]. Continuity of carer fosters a genuine woman‐midwife relationship that can engender a sense of control for the woman, making it more likely she will access MLBS [33].

### 4.7 Influence and Beliefs About Birth

A significant factor in the choice of place of birth is the woman's cultural norms, in some cases influenced by older women in the community [60, 66]. Some first‐generation migrant women, including of Pakistani or Bengali origin, placed a particular value on hospital‐based, doctor‐led obstetric care as safe and modern. These migrant women then perceive UK based MLBS as less advanced, less safe, carrying a stigma, or associated with higher mortality rates in ‘the village’ in the origin country [59, 60, 64]. Even after one or two generations, this influence was significant, particularly so in studies related to women from Pakistani and Bengal backgrounds [59, 60, 64]. For some women from Bengali communities, it created a burden of choice about possible blame if anything did go wrong, leading them to keep their choice for a MLBS from their families [59]. One emerging point was the female‐only nature of MLBS, which echoed the positive aspects of their foremothers' births in Bangladesh as safe from undesirable attendance by male healthcare professionals [59, 64].

When making choices that diverged from family expectations, membership of antenatal groups and knowing someone in the community who had given birth there were significant factors in choosing a MLBS, especially if the woman heard their birth story [30, 59, 62]. Some women found that wider social media gave them access to networks around physiological birth, water birth, and MLBS. Tours of the MLU helped reassure and enabled some women to be the first in their community to choose an MLBS. Representation in the form of photos and birth stories of women of the same ethnicity displayed in the MLU building and posted on social media pages was a positive factor in normalizing the choice [59, 60].

As a result of a risk‐averse medical culture and media influences, both midwives and women can have a perception of MLBS as ‘risky’ despite strong evidence to the contrary [21, 24], deterring midwives from offering it as a genuine choice [45, 60, 61, 64, 67]. Midwives can feel caught between woman‐centered choice and the tension of professional accountability, exacerbated when negotiating unfamiliar cultural practices [45, 61, 64]. Goodwin et al. [64] interviewed midwives who believed Pakistani women would be less likely to seek medical help due to religious beliefs, although they noted that good relationships with women reduced prejudice. Foley et al. [50] and Naylor Smith et al. [62] discuss the issue of living in large extended families as a barrier to choosing homebirth, although both note this was not the case for everyone.

### 4.8 Midwife‐Led Birth Settings as Empowering

Racialized women being pleasantly surprised by the MLU environment was reported by McCourt et al. [60], Reeve Jones [59, 63], and Rayment et al. [45] Racialized women felt treated in a way that they did not normally experience: as special, accessing a luxury akin to a spa or like royalty [45, 60]. "I felt like a princess. Maybe that's how Kate Middleton and them lot get treated when they give birth in their private hospitals. But it wasn't private. I didn't pay anything for it, but the service was just first class honest (p25) [59]. Women found the MLU calm, clean and ‘*absolutely fantastic*’ [63] (p290) and choose it as a place they received respect and kindness [59].

Women who have a first birth at a MLU tend to have subsequent births there and to influence other women in their communities, viewing it as safe and straddling both physiological birth and access to obstetric care if needed [59, 62, 63]. The sense of pride in forging a new path and choosing a MLBS became a significant part of some women's identities, different from their mothers and grandmothers, including questioning the medical professionals and making empowered decisions [59].

## 5 Discussion

### 5.1 Statement of Principal Findings

There is a sparsity of existing literature on the factors affecting access to MLBS for racialized women. Of the 14 articles we found with any reference to the theme, only one specifically addressed the question. Nevertheless, we developed some clear themes. There is reported bias in information given by midwives regarding place of birth choices and evidence of gaps in professional provision of accurate evidence‐based information. There are some system‐level barriers, such as admission criteria. For some in the studies, community beliefs about birth and cultural norms played a part, at times conflicting with recent evidence‐based information showing MLBS as able to provide safe, women‐centered care.

### 5.2 Strengths and Weaknesses of This Review

The strength of this scoping review is that it takes a specifically midwifery lens to the problem of racial inequality in birth and place of birth. The main limitation was the lack of material directly related to our question, with most of the research used containing minimal reference to our central question. As it was not the focus of the selected research, it makes the conclusions somewhat rhizomatic. A second limitation was most of the research focusing on women already classified as ‘low‐risk’ as we discuss further below. Thirdly is the issue of using the broad category of ‘racialized women’. While it is useful to identify common structural issues, there is a risk of implying homogeneity and overgeneralizing. Finally, it could be that local or grassroots innovations are taking place that were not revealed in our searches due to the material being less widely publicized.

### 5.3 Review Findings in the Context of Existing Research and UK Policy

Most research on MLBS, including the studies used in this scoping review, focuses on place of birth for ‘low‐risk’ women only. This is despite the fact that the Birthplace Study showed that women with ‘intermediate’ risk factors who had home births showed comparable neonatal outcomes and better maternal outcomes compared to women with the same intermediate risk factors birthing in an obstetric unit [69, 70]. It is important to note that how women become classified as ‘high‐risk’ is historically and geographically specific and may have a racialized aspect. Most research on the higher proportion of racialized women classified as ‘high‐risk’ focuses on the effect of allostatic load or ‘weathering’ and the correlation of race with lower socioeconomic status [11, 71, 72]. However, it is possible that racialized women may be more likely, compared to White women, to be treated as ‘high‐risk’ when they have ‘intermediate’ factors that could have relatively good outcomes in MLBS. Additionally, seeing White women's and White babies' bodies as the ‘norm’ can risk pathologizing what is normal, and, conversely, missing what is pathological for racialized women and their babies—for example, the problems of standard BMI parameters, or neonatal APGAR scores and jaundice recognition based on White populations [73, 74]. These factors could contribute to explaining both a lower use of MLBS by racialized women and the (related) higher medical intervention rates among these women.

Our review echoes the NHS Race and Health Observatory's 2022 report [75] concluding with the role of local hubs, the need to focus on communities and institutions rather than individual solutions alone, and the need to involve women from ethnic minorities in the co‐production of interventions and research. Unlike obstetric settings, midwifery services and MLBS *can* be geographically and culturally situated in the community. The House of Commons Women and Equalities Committee on Black Maternal Health [71] emphasizes professional bias and racism, and promotes staff training as a part of the solution. Similarly, the UK's Maternity Transformation Programme places emphasis on personalized care for all [76]. Our review shows the importance of both specific interventions embedded in communities of racialized women and the unique role midwife‐led care and MLBS can play in redressing balance. The power relations and hierarchy inherent in the NHS organization, the health issues, and the medical model as outlined by Black British feminists, such as Bryan et al. [77], come into sharp focus regarding racialized women's access to MLBS. What is unique about our report is the emphasis on engaging in women‐centered biopsychosocial care, thus having a higher chance of offering care from a genuine ‘midwifery standpoint’ [78]. This relational care may lead to improved experiences and possibly improved outcomes for racialized women. Group antenatal care, by relocating authoritative knowledge back to the women, with facilitative midwifery and peer support, is particularly important for those who have been at the sharp end of dehumanizing and disempowering medical practice as individuals and with a cultural legacy of systemic racism [66].

## 6 Implications for Policy

Making MLBS accessible for all women is the first step to making them accessible for racialized women. This could include increased provision and information, decision‐making aids, staff training, and institutional support for midwife‐led care [13, 42, 71, 79, 80]. An ‘opt‐out’, or defaulting to a MLBS, for women without biomedical risk factors, with full discussion about options of obstetric‐led care in the event of clinical need or maternal choice, could remove the barriers of biased information giving [60]. Home assessments in early labor with the place of birth not fixed prior to that point could also remove the barrier of defaulting to the obstetric unit [30].

To overcome bias and structural inequality, equal access for racialized women requires additional measures. Community outreach, including to older generation women, could help shift the dominant discourse within communities to reflect the safety and comfort of MLBS [64]. An increase in MLBS use and the sharing of stories normalizes MLBS and increases the communities' knowledge and confidence in MLBS and in women's physiology and capabilities [67]. Representation in the form of pictures and accessible information about MLBS may help with women's and midwives' assumptions about who such services are for [30, 59, 81].

Our review showed that better midwife‐women relations in the antenatal period may lead to increased access for racialized women to MLBS. Therefore, services with time and flexibility may have a positive impact, as might Public Health England's aim to improve outcomes for racialized women through midwifery‐led continuity of carer [82].

Situating MLBS within settings used by racialized communities may increase access by providing visibility and a sense of familiarity. Long‐term integrated community outreach, along with opt‐out models and education for midwives, may go some way to addressing the problem.

## 7 Need for Future Research

The paucity of data we found indicates the need for robust research focusing specifically on the question of racialized women's access to MLBS, both in terms of the barriers and the possible solutions. The results of this research could help increase access to MLBS, thus engendering a shift from hierarchical to relational care and hopefully improving outcomes and experiences for racialized women. Risk classifications and MLBS criteria are also areas that merit future research. A review of risk classifications and MLBS admission criteria and a move away from a ‘high‐risk’/‘low‐risk’ binary may be of benefit.

## Conflicts of Interest

The authors declare no conflicts of interest.

## Data Availability

The data that support the findings of this study are openly available in [repository name] at [URL], reference number [reference number]. All the articles used for this review are available via academic databases.
